# A Critical Role for CD8 T Cells in a Nonhuman Primate Model of Tuberculosis

**DOI:** 10.1371/journal.ppat.1000392

**Published:** 2009-04-17

**Authors:** Crystal Y. Chen, Dan Huang, Richard C. Wang, Ling Shen, Gucheng Zeng, Shuyun Yao, Yun Shen, Lisa Halliday, Jeff Fortman, Milton McAllister, Jim Estep, Robert Hunt, Daphne Vasconcelos, George Du, Steven A. Porcelli, Michelle H. Larsen, William R. Jacobs, Barton F. Haynes, Norman L. Letvin, Zheng W. Chen

**Affiliations:** 1 Department of Microbiology and Immunology, Center for Primate Biomedical Research, University of Illinois College of Medicine, Chicago, Illinois, United States of America; 2 Harvard Medical School, Beth Israel Deaconess Medical Center, Boston, Massachusetts, United States of America; 3 BRL, University of Illinois, Chicago, Illinois, United States of America; 4 Department of Pathobiology, College of Veterinary Medicine, University of Illinois, Urbana-Champaign, Illinois, United States of America; 5 Battelle Medical Research Evaluation Facility, Battelle Memorial Institute, Columbus, Ohio, United States of America; 6 Department of Microbiology and Immunology, Albert Einstein College of Medicine, Bronx, New York, United States of America; 7 Howard Hughes Medical Institute and Albert Einstein College of Medicine, Bronx, New York, United States of America; 8 Duke Human Vaccine Institute, Duke University, Durham, North Carolina, United States of America; Johns Hopkins School of Medicine, United States of America

## Abstract

The role of CD8 T cells in anti-tuberculosis immunity in humans remains unknown, and studies of CD8 T cell–mediated protection against tuberculosis in mice have yielded controversial results. Unlike mice, humans and nonhuman primates share a number of important features of the immune system that relate directly to the specificity and functions of CD8 T cells, such as the expression of group 1 CD1 proteins that are capable of presenting *Mycobacterium tuberculosis* lipids antigens and the cytotoxic/bactericidal protein granulysin. Employing a more relevant nonhuman primate model of human tuberculosis, we examined the contribution of BCG- or *M. tuberculosis*-elicited CD8 T cells to vaccine-induced immunity against tuberculosis. CD8 depletion compromised BCG vaccine-induced immune control of *M. tuberculosis* replication in the vaccinated rhesus macaques. Depletion of CD8 T cells in BCG-vaccinated rhesus macaques led to a significant decrease in the vaccine-induced immunity against tuberculosis. Consistently, depletion of CD8 T cells in rhesus macaques that had been previously infected with *M. tuberculosis* and cured by antibiotic therapy also resulted in a loss of anti-tuberculosis immunity upon *M. tuberculosis* re-infection. The current study demonstrates a major role for CD8 T cells in anti-tuberculosis immunity, and supports the view that CD8 T cells should be included in strategies for development of new tuberculosis vaccines and immunotherapeutics.

## Author Summary

Tuberculosis, HIV/AIDS and malaria remain top killers worldwide. Cell-mediated immune responses play a crucial role in immunity against tuberculosis. While CD4 T cells are well described for their protection against tuberculosis, little is known about the role of human CD8 T cells in anti-tuberculosis immunity. Studies done to date in mice have yielded conflicting results regarding the role of mouse CD8 T cells in tuberculosis. Since there are considerable differences in CD8 T cell biology between mice and primates including humans and macaques, studies in humans or macaques are crucial for clarifying human CD8 T cell–mediated immunity against tuberculosis. Thus, we used a macaque tuberculosis model to examine the contribution of CD8 T cells to vaccine-induced immunity against tuberculosis. We found that CD8 T cells play a role in BCG vaccine-induced immune control of *Mycobacterium tuberculosis* replication and in the vaccine-induced immunity against tuberculosis. Consistently, memory CD8 T cells also play a crucial role in anti-tuberculosis immunity upon *M. tuberculosis* re-infection. The findings in the current study provide evidence that human CD8 T cells are of importance for anti-tuberculosis immunity, and support the view that CD8 T cells should be targeted for development of new tuberculosis vaccines and immunotherapeutics.

## Introduction

Tuberculosis remains one of the major causes of global mortality, and has become increasingly prevalent and deadly as a result of HIV/AIDS pandemic and the emergence of extensively drug resistant (XDR) strains of *M. tuberculosis* [1]. Elucidating the relevant components of anti-tuberculosis immunity is therefore of critical importance and urgency to facilitate the development of safe and effective vaccines for the global battle against tuberculosis. The attenuated *Mycobacterium bovis* strain Bacille Calmette-Guerin (BCG) is currently the sole vaccine for tuberculosis that is approved for use in humans. BCG vaccination has been shown to induce protection against severe tuberculosis in children and nonhuman primates, but it is inconsistent or ineffective at conferring protection against tuberculosis in adults who have been vaccinated early in life [2],[3],[4],[5],[6].

Although it is widely accepted that CD4 T cells play a critical role in the ability of humans and experimental animals to resist active *M. tuberculosis* infection [7], the contribution of CD8 T cells to natural or BCG-induced immunity against tuberculosis remains unclear. Some *in vivo* studies in mice and bovines or *in vitro* work in humans provide evidence supporting the contribution of CD8 T cells to immunity against tuberculosis [8],[9],[10],[11],[12],[13],[14],[15],[16],[17],[18], whereas a number of studies in mouse models of tuberculosis have argued against a significant role for CD8 T cells in the control of primary *M. tuberculosis* infection [19],[20],[21],[22],[23],[24],[25]. However, it is likely that the mouse model of tuberculosis does not accurately reflect the complete picture of how protective immune responses against tuberculosis develop in humans, and better models of this disease are needed to enable a full understanding of this process. We propose that nonhuman primates should provide more relevant models for evaluating a role of human CD8+ T cells in BCG vaccine-induced immunity against tuberculosis [6],[26],[27],[28],[29],[30]. Unlike mice and other rodents studied to date, rhesus macaques share with humans a number of important features of the immune system that relate directly to the specificity and functions of CD8 T cells, such as the expression of group 1 CD1 proteins that are capable of presenting *M. tuberculosis* lipids antigens and the cytotoxic/bactericidal protein granulysin [31],[32]. Importantly, we and others have shown that disease course and pathology in *M. tuberculosis*-infected macaques resemble human tuberculosis, and that vaccine-induced immunity to tuberculosis can be experimentally evaluated in rhesus and cynomolgus macaques [6],[28],[29],[33],[34]. Although the importance of CD4 T cells in nonhuman primate models of tuberculosis has been reported, data are currently lacking on the role of CD8 T cells [35],[36].

## Results

### Anti-CD8 Ab treatment of BCG-vaccinated rhesus macaques induced profound depletion of CD8 lymphocytes and BCG-elicited CD8 T cells during pulmonary *M. tuberculosis* infection

**Figure 1:**
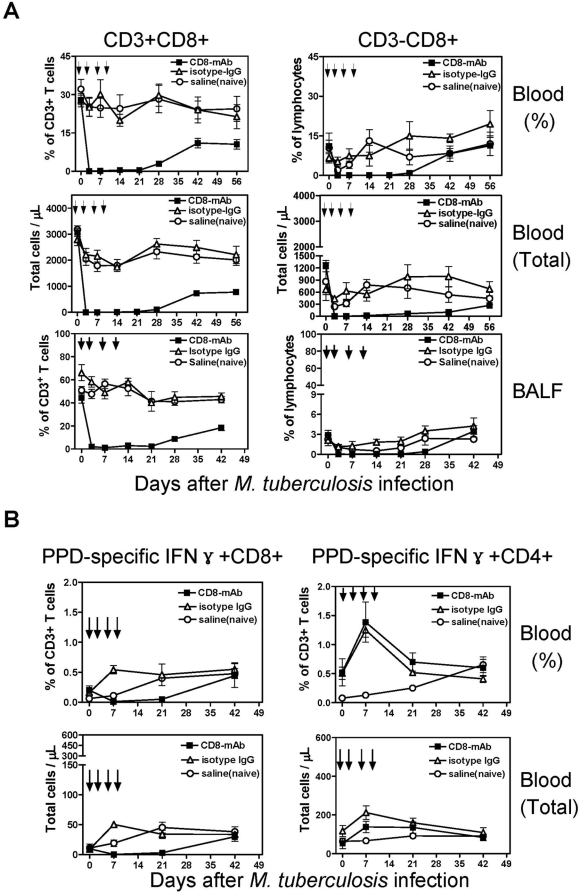
CD8 Ab treatment of BCG-vaccinated macaques resulted in profound depletion of CD8 lymphocytes and mycobacterium-specific CD8 T effector cells during *M. tuberculosis* infection. (A) Flow cytometry data showed that anti-CD8 Ab-treated BCG-vaccinated macaques, but not isotype control IgG-treated BCG-vaccinated or naïve saline-treated macaques, exhibited marked decreases in percentages and absolute (data not shown) numbers of CD3+CD8+ and CD3−CD8+ lymphocytes in blood (upper left) and BAL fluid (upper right) during *M. tuberculosis* infection. Means for six animals are shown for each group, and error bars represent SEM. Arrows indicate the times for the treatment with anti-CD8 Ab or control antibodies. (B) Intracellular cytokine staining data showed that anti-CD8 Ab-treated BCG-vaccinated macaques exhibited an absence of PPD-specific IFNγ-producing CD8 T cells early after *M. tuberculosis* infection.

To directly examine the importance of CD8 T cells in BCG vaccine-induced anti-tuberculosis immunity, six BCG-vaccinated macaques were treated with depleting anti-CD8 antibody, cM-T807 [37] at the same time that they received pulmonary inoculation with *M. tuberculosis*. Pulmonary *M. tuberculosis* infection was introduced by bronchoscope-guided inoculation of 3000 CFU of *M. tuberculosis* into the right caudal lobe [29]. As controls, six BCG-vaccinated moneys were treated similarly with isotype-matched human IgG at the time of infection with *M. tuberculosis*, and six unvaccinated macaques (naïve controls) were treated with saline at the time pulmonary *M. tuberculosis* was introduced. Treatment with depleting anti-CD8 Ab resulted in profound depletion of CD8 cells during *M. tuberculosis* infection, with CD3+CD8+ T cells and CD3−CD8+ cells falling to barely detectable levels in blood and BAL fluid at 1–4 weeks after the simultaneous anti-CD8 Ab treatment and *M. tuberculosis* infection (Fig. 1A). Even at 7 weeks after the anti-CD8 Ab treatment, CD8 T cells remained partially depleted (Fig. 1A). Consistently, peptide antigen-specific IFNγ-producing CD8 T effector cells also became undetectable early after the simultaneous CD8 depletion and *M. tuberculosis* infection (Fig. 1B). In contrast, the macaques treated with the nonspecific isotype matched control Ab exhibited increased percentages and numbers of CD8 T cells, including peptide-specific CD8 T effector cells, after the *M. tuberculosis* infection (Fig. 1A,1B). Thus, we concluded that this method of CD8 T cell depletion was suitable for evaluating the impact of previously immunized CD8 T cells on the progression and outcome of pulmonary tuberculosis infection in the macaque model.

### CD8 depletion compromised BCG vaccine-induced immune control of *M. tuberculosis* replication in the vaccinated macaques

**Figure 2:**
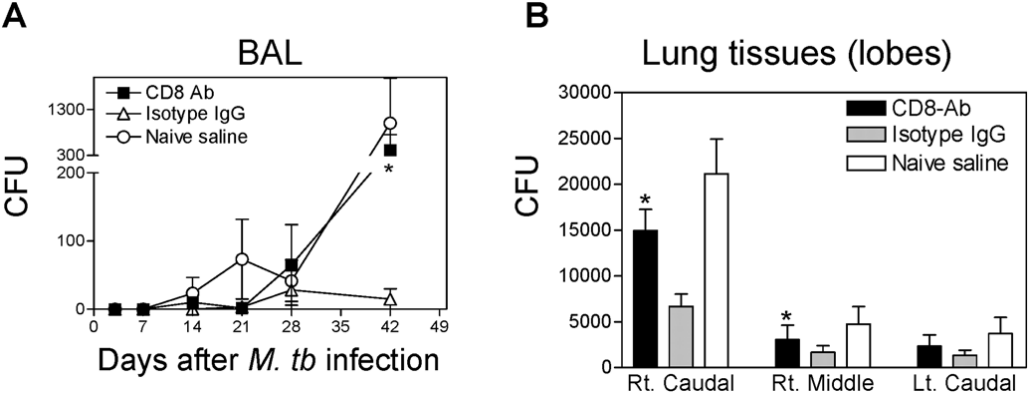
Depletion of CD8 lymphocytes resulted in a significant decrease in BCG-induced immune control of *M. tuberculosis*. (A) CD8 Ab-treated macaques exhibited higher levels of bacilli in BAL fluid than isotype IgG-treated macaques after pulmonary *M. tuberculosis* infection. Data are means +/− SEM of CFU counts/10 ml BAL fluid derived from 6 macaques for each group; * indicates p<0.05 for a comparison between the CD8 Ab-treated and isotype IgG-treated group. (B) CD8 Ab-treated macaques showed significantly higher levels of bacilli organisms in lung tissues than isotype control macaques after pulmonary *M. tuberculosis* infection. Data are mean values with SEM error bars of CFU counts/1 ml lung tissue homogenates derived from 6 macaques for each group. Rt, right; Lt, left. Note that right caudal lobe was the *M. tuberculosis* infection site for each macaque; *, p<0.05 (by both ANOVA and nonparametric *t* test) for a comparison between the CD8 Ab-treated and isotype control IgG-treated groups.

To determine the impact of depletion of CD8 lymphocytes including BCG-elicited CD8 T cells on immunity to tuberculosis in the rhesus macaque model, we evaluated four aspects of vaccine efficacy: (i) severe clinical manifestations such as marked changes in temperature, progressive coughing, dispnea/distress, anorexia, altered consciousness or weight loss during a 2-month follow-up; (ii) bacterial colony counts in BAL fluid at various times after *M. tuberculosis* infection and in lung tissues at necropsy; (iii) gross pathology of the lungs and other thoracic and extrathoracic organs at necropsy; (iv) histologic evaluation of tissues for granulomas and other microscopic lesions. None of the macaques in any of the control or treatment groups developed fatal clinical tuberculosis or significant loss of body weight during 2-months of follow-up. This was consistent with earlier reports that Chinese rhesus macaques were more resistant to infections than Indian rhesus macaques, and that adult animals were less susceptible to fatal tuberculosis than juveniles [6],[27],[38]. Nevertheless, the CD8 Ab-treated group of BCG-vaccinated macaques showed significantly higher bacterial counts in BAL fluids than the IgG isotype control group at 42 days after *M. tuberculosis* infection (Fig. 2A). In addition, the CD8 Ab-treated group also had significantly higher bacterial CFU counts in the lung tissues in the right caudal lobe (*M. tuberculosis* infection site) and distant right middle lobe than the isotype IgG-treated group (Fig. 2B). These results suggested that CD8 T cells contributed to BCG vaccine-induced immune control of *M. tuberculosis* replication in the vaccinated macaques.

### Depletion of CD8 T cells in BCG-vaccinated rhesus macaques led to a significant decrease in the vaccine-induced immunity against tuberculosis

**Figure 3:**
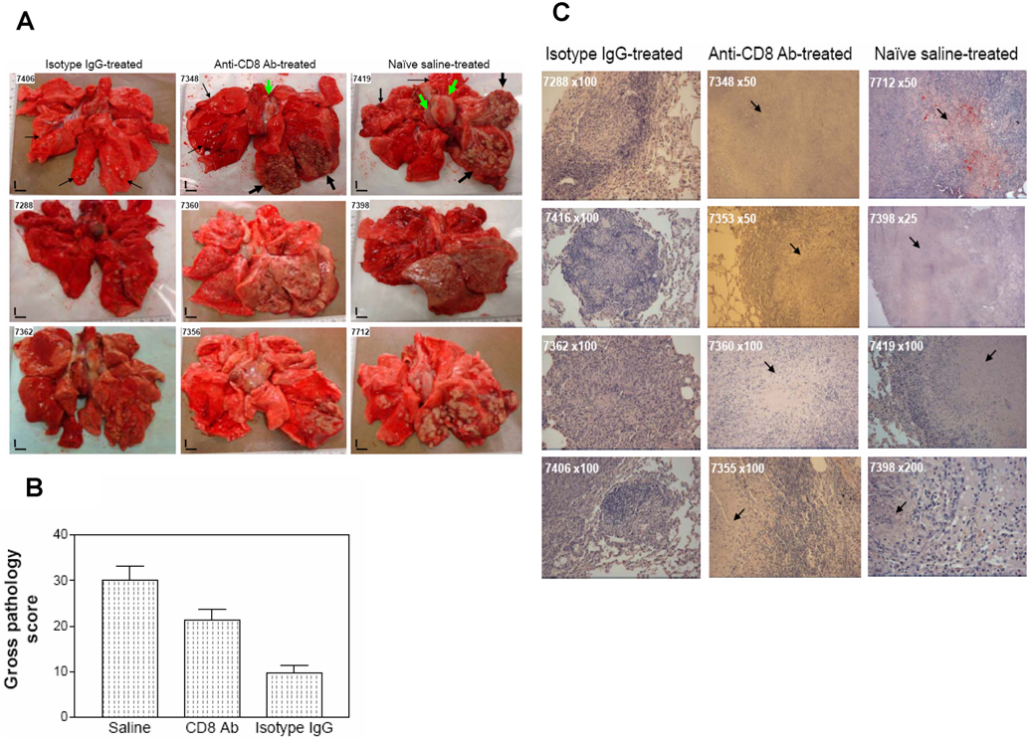
Depletion of CD8 lymphocytes in CD8 Ab-treated macaques resulted in a significant decrease in BCG vaccine-induced immunity against tuberculosis after *M. tuberculosis* infection. (A) Top-panel photos show that the isotype IgG-treated BCG-vaccinated macaque (7406) exhibited limited numbers of granulomas (small arrows) in the cut-section of the right caudal lobe; the CD8 Ab-treated macaque (7348) showed extensive tuberculosis granulomas (large arrows) in the right caudal lobe, with a unilaterally enlarged hilar lymph node (a large green arrow). A naïve macaque (7419) showed large extensive tuberculosis lesions with caseation (large arrows), and much larger bilateral hilar lymph nodes (large green arrows). Middle- and bottom-panel photos show tuberculosis lesions in cut-sections of lungs of six monkeys in three respective groups. Extent and severity of the lesions could be adjudged based on the examples pointed by arrows at the top panel photos. Overall, CD8 Ab-treated macaques exhibited greater numbers of lung lobes displaying extensive coalescing granulomas than isotype IgG-treated control animals (p<0.05, by nonparametric *t* test). The CD8 Ab-treated macaques also showed more lobes with extensive caseating and miliary lesions or caseation pneumonia than the isotype IgG-treated controls (p<0.05, by nonparametric *t* test). Tuberculosis lesions in the CD8 Ab-treated macaques were more likely distributed or disseminated in other lobes or opposite lungs and hilar nodes/pleural. Small vertical/horizontal bars at bottom-left corner of each photo represent the 1-cm scale derived from the fluorescence rulers of each original photo. (B) Gross pathology scores of tuberculosis lesions in CD8 Ab-treated group, Isotype control IgG-treated group and naïve saline-treated group of macaques. Each macaque was given a total gross pathology scores based on the tuberculosis lesions in thoracic and extrathoracic organs. The mean gross pathology score was then calculated for each group of macaques and subjected to statistical analysis. *, P<0.05 (by ANOVA and nonparametric *t* test) for comparison between CD8 Ab-treated and isotype control IgG-treated groups, and for comparison between CD8 Ab-treated and naïve groups. (C) Histology evaluation of lung tissue sections of CD8 Ab-treated, isotype control IgG-treated and naïve saline-treated macaques. Shown are H&E stained sections taken from four representative macaques for each group, with macaque ID and magnification indicated for each image. Note that the isotype control IgG-treated macaques exhibited well-contained granulomas, which were generally infiltrated by numerous lymphocytes and some neutrophils. The CD8-depleted macaques displayed less-contained granulomas that were more likely to be necrotic (arrows). Naïve macaques showed less lymphocytic and more necrotic tuberculosis lesions than the other two groups.

The next critical question regarding the contribution of CD8 T cells to BCG vaccine-induced immunity against tuberculosis was whether depletion of CD8 lymphocytes in BCG-vaccinated macaques led to more severe tuberculosis lesions. To address this, we performed complete necropsy studies 2 months after *M. tuberculosis* infection of three groups of macaques. The gross pathology was evaluated in detail in a quantitative fashion using a previously described scoring systems [6],[28],[29],[33], and then compared among the three groups of *M. tuberculosis*-infected macaques. The isotype control IgG-treated BCG-vaccinated macaques exhibited apparent protection against tuberculosis compared to the naïve saline-treated group (Fig. 3). For these macaques with intact BCG-elicited CD8 T cells, *M. tuberculosis* infection appeared to be well contained by individual granulomas predominantly at the primary infection site in the right caudal lobe (Fig. 3A). In contrast, the CD8 Ab-treated group of BCG-vaccinated macaques exhibited a reduced containment of tuberculosis lesions in the infection site. CD8 Ab-treated macaques showed greater numbers of lung lobes showing extensive coalescing granulomas than isotype IgG-treated control animals (p<0.05, Fig. 3A). These CD8-depleted macaques also exhibited more lobes with severe/extensive caseating and miliary lesions or caseation pneumonia than the isotype IgG-treated controls (p<0.05, Fig. 3A). Tuberculosis lesions in the CD8 Ab-treated macaques were more likely distributed or disseminated in other lobes or opposite lungs and hilar lymph nodes/pleural (Fig. 3A). Furthermore, depletion of CD8 lymphocytes led to systemic dissemination of tuberculosis, as grossly apparent granulomas were identified in extra-thoracic organs in five of 6 macaques treated with anti-CD8 Ab. When total scores of gross tuberculosis lesions were calculated for individual macaques, and compared among the three groups of animals, we found that the CD8 Ab-treated group developed significantly worse gross tuberculosis lesions than the isotype IgG-treated group (p<0.05, Fig. 3B).

The severity of tuberculosis following depletion of CD8 lymphocytes was further evaluated at the microscopic level, which revealed that depletion of CD8 lymphocytes during *M. tuberculosis* infection led to changes in the organization and cellular composition of granulomas. In the isotype control IgG-treated macaques with intact CD8 T cells, *M. tuberculosis* infection was usually contained by well-organized granulomas infiltrated by numerous lymphocytes and some neutrophils (Fig. 3C). However, in the CD8 Ab-treated macaques, lymphocytic infiltration was reduced and necrosis was more pronounced, especially in the lungs (Fig. 3C). Furthermore, microscopic scanning of a series of tissue sections derived from extrathoracic organs revealed that five of six CD8 Ab-treated BCG-vaccinated macaques had a number of granulomatous lesions in the spleen, whereas only one of six isotype control IgG-treated macaques displayed microscopic granulomas in the spleen. All naïve macaques developed multiple granulomatous lesions in spleens and livers (Fig. 3C).

### Depletion of CD8 T cells in macaques that had been previously infected with *M. tuberculosis* and cured by antibiotic therapy also resulted in a loss of anti-tuberculosis immunity upon *M. tuberculosis* re-infection

**Figure 4:**
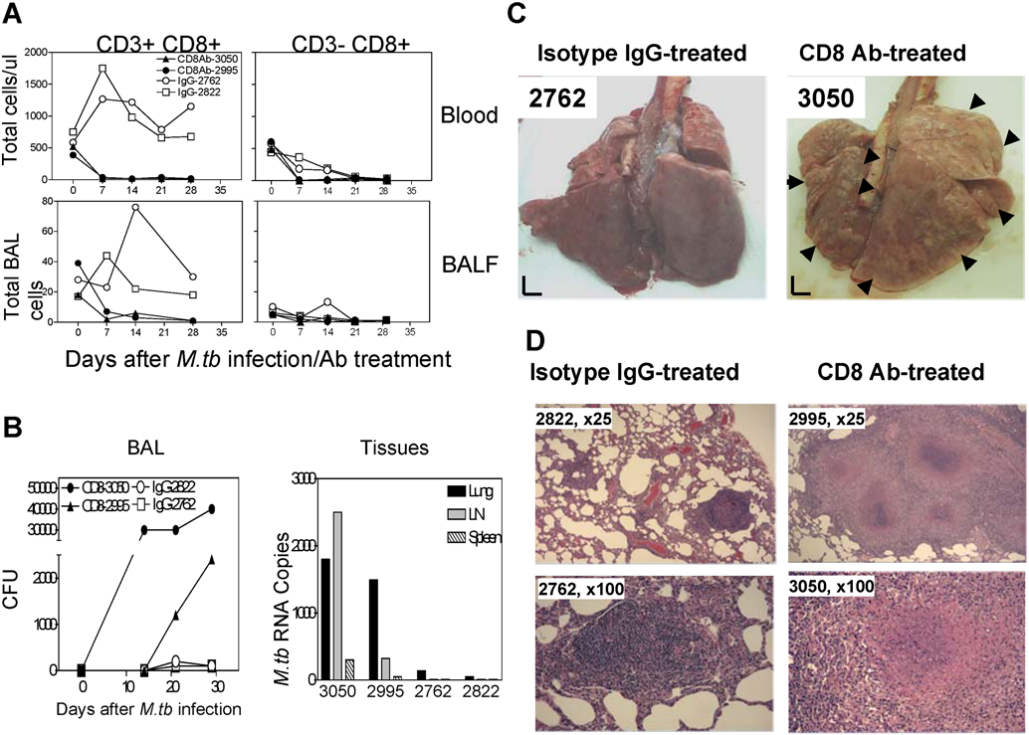
Depletion of CD8 lymphocytes in macaques immunized by previous *M. tuberculosis* infection resulted in a loss of immune control of tuberculosis following re-infection. (A) Anti-CD8 Ab treatment of the *M. tuberculosis*-immunized macaques resulted in profound depletion of CD8 lymphocytes in *M. tuberculosis* re-infection. Shown are absolute levels of blood CD8 cells (/ul) and BAL fluid CD8 cells (×50,000). (B) Depletion of CD8 lymphocytes in the *M. tuberculosis*-immunized macaques led to higher levels of bacilli in BAL fluids and increased *M. tuberculosis* RNA in tissues following *M. tuberculosis* re-infection by aerosol. Numbers of bacilli are shown as CFU counts in 10 ml of BAL fluid; *M. tuberculosis* RNA was determined as Ag85B RNA copy numbers in 10 mg of tissue [6],[29],[36]. (C) The CD8 Ab-treated macaques with CD8 depletion developed severe forms of tuberculosis after *M. tuberculosis* re-infection by aerosol. Macaque ID are indicated for CD8 Ab-treated (right) and isotype control IgG-treated (left) macaques. Shown are the lung surface images of two representative macaques (partially cut in the lung of macaque 2762). Note that the CD8 Ab-treated macaques exhibited pale-colored lungs with dissemination of >0.5 cm coalescing or caseating granulomas or tubercle nodules (the lesion-containing areas are pointed out by large arrows on surface view from 3050) and apparent caseation necrosis. The control macaques displayed no or few small non-caseating granulomas as indicated by small arrows in the lungs. When >0.5 cm coalescing or caseating tubercle nodules both lungs were scored using the scoring system as described in Methods and [41] for the individual macaques, the pathology scores (mean±SD = 72±24) of the CD8-depleted macaques were significantly worse than those (mean±SD = 6±2) of the control animals (p<0.01, by nonparametric *t* test). (D) Histology of representative granulomas seen in CD8 Ab-treated (right panel) and isotype control IgG-treated (left panel) macaques. Shown are H&E stained lung sections, with macaque ID and magnification indicated in each slide. Note that granulomas in isotype IgG-treated macaques were small, and highly lymphocytic without apparent necrosis. Granulomas in CD8 Ab-treated macaques were large and less lymphocytic, with necrosis seen in the center as pointed out by arrows.

Finally, we sought to examine whether CD8 memory T cells resulting from prior *M. tuberculosis* infection were also important for anti-tuberculosis immunity. This question is directly relevant to the development of novel vaccines that are based on attenuated *M. tuberculosis* mutants with virulence gene deletion or other modifications [39],[40], and also is important for understanding the potential immunological defects that allow re-infection with *M. tuberculosis* and reactivation of latent tuberculosis. To generate *M. tuberculosis*-immunized animals with anti-tuberculosis immune memory, we made use of BCG-vaccinated juvenile rhesus macaques who exhibited rapid recall T cell responses and survived fatal tuberculosis for 2.5 months after aerosol challenge with 400 CFU *M. tuberculosis*. Under these conditions, unimmunized naïve control macaques consistently became moribund due to development of early fatal tuberculosis [6]. These *M. tuberculosis*-exposed macaques that had transient low levels of bacillus but no evidence of active tuberculosis after the challenge were treated daily for 3 months with a regimen of anti-tuberculosis drugs (5 mg/kg Isoniazid plus and 15 mg/kg Pyrazinamide), and then rested for 2 months during which they showed no evidence of detectable bacilli in repeated lung washings or of clinical tuberculosis. Two animals were then treated with depleting anti-CD8 Ab, and two with isotype control IgG at the time of re-infection with 3000 CFU *M. tuberculosis* by aerosol. The CD8 Ab-treated but not isotype IgG-treated macaques showed profound depletion of CD8 lymphocytes in the blood and BAL fluid (Fig. 4A). Importantly, the CD8 Ab-treated macaques with CD8 depletion showed much higher levels of *M. tuberculosis* burdens in BAL fluid and lung tissues than the IgG-treated control macaques (Fig. 4B). The gross pathology studies showed the isotype IgG-treated macaques displayed no or limited numbers of small non-caseating granulomas (Fig. 4C). In contrast, the CD8-depleted macaques showed dissemination of >0.5 cm coalescing or caseating granulomas or tubercle nodules in both lungs (Fig. 4C). When such tuberculosis lesions were scored using the scoring system as described [41] in these individual macaques, the lesions scores of CD8-depleted macaques were significantly worse than the isotype IgG-treated controls (p<0.01, by nonparametric *student t* test). Consistently, histologic analyses revealed that granulomas in the CD8 Ab-treated macaques with CD8 depletion were large and necrotic in the center, whereas those in the isotype IgG-treated macaques with CD8 intact were small and highly lymphocytic without apparent necrosis (Fig. 4D). These results from *M. tuberculosis*-immunized, drug-treated macaques provided novel evidence suggesting that *M. tuberculosis*-elicited CD8 T cells are important for memory protection against tuberculosis after *M. tuberculosis* re-infection.

## Discussion

Our findings in BCG-vaccinated and *M. tuberculosis*-immunized macaques strongly support the hypothesis that CD8 T cells play a critical role in host defense against tuberculosis despite that the role of CD3−CD8+ cells cannot be completely ruled out. Although the depletion of other cells expressing CD8 in addition to CD8 memory T cells could potentially have contributed to the observed effects of anti-CD8 treatment, this seems unlikely for several reasons. First of all, the anti-CD8 effect was manifested as a loss of immunological memory induced by previous BCG immunization or *M. tuberculosis* infection, and such memory is a characteristic of CD8 T cells but not of other cells known to express CD8 such as NK cells. In addition, most macaque dendritic cells, like their human counterparts, do not express CD8 [42], and thus immune responses of these cells in tuberculosis might not be affected directly by the antibody-mediated CD8 depletion. Furthermore, depletion of a subset of NK cells and potentially other CD8+ cells that do not express TCR/CD3 was at most partial by the anti-CD8 treatment in contrast to the depletion of CD8 T cells which was essentially complete for 3–4 weeks after infection. The memory CD8 T cells conferring anti-tuberculosis immunity in BCG- and *M. tuberculosis*-immunized macaques may include at least three different CD8 T cell subpopulations: (i) peptide-specific classical MHC class I-restricted CD8 T cells; (ii) non-classical MHC class I-restricted CD8 T cells [43]; (iii) lipid-specific CD1-restricted CD8 T cells [44]. They may also include phosphoantigen-specific Vγ2Vδ2 T cells, the γδ T-cell subset existing only in primates, since macaque Vγ2Vδ2 T cells can express CD8 and exhibit anti-microbial responses during clonal activation/expansion [45]. The relative importance and protective surrogate markers of these different CD8 T cell subpopulations to anti-tuberculosis immunity is currently not known. However, it is noteworthy that only the first of these three populations is present in mice, whereas all three are present in humans. For this reason, studies such as those reported here in nonhuman primates may reveal important activities of CD8 T cells that are not apparent in mouse models of tuberculosis.

Our studies add to a growing body of evidence that supports the view that CD8 T cells are of critical importance for effective immunity against *M. tuberculosis*. *Mycobacterium*-specific CD8 T cells have been shown to possess effector functions that are likely to assist in the containment and clearance of *M. tuberculosis*, including cytolytic effector function, cytokine production and direct antimicrobial activity through the production of anti-microbial peptides such as granulysin [31],[46],[47]. Even in mice and other rodents which may lack some of the important recognition and effector activities of CD8 T cells in primates, it is noteworthy that studies have shown that approaches to vaccination that augment CD8 T cell responses can provide better protection against challenge with *M. tuberculosis* than what is achieved with standard BCG vaccination [48]. Such findings, together with the results of the current study, strongly suggest that CD8 T cells should be considered as relevant targets in the design and development of new tuberculosis vaccines and immunotherapeutics.

## Methods

### Macaque animals

A total of 22 monkeys were included in these studies. 18 Chinese rhesus (*Macaca mulatta*), 4–7 years old, were used to assess CD8 T cells for BCG vaccine-induced immunity; 4 juvenile Indian rhesus monkeys, 2.9 years old, were used for evaluating *M. tuberculosis*-immunized CD8 T cells. Studies using all the animals were documented in animal protocols and approved by IACUC.

### BCG vaccination

12 Chinese rhesus monkeys were vaccinated intradermally with 50×10^6^ CFU of BCG Danish (FDA stock), as previously described [33]. 4 months after the vaccination, the monkeys were randomly assigned to CD8 Ab- and isotype IgG-treated groups.

### *M. tuberculosis* immunization and anti-tuberculosis drug treatment

Four Indian rhesus monkeys were first vaccinated with BCG, and then challenged with 400 CFU of *M. tuberculosis* by aerosol as previously described [6]. These vaccinated monkeys survived fatal tuberculosis during a 2.5-month follow-up, whereas four control naïve animals became moribund and had to be euthanized due to the development of fatal tuberculosis within 1.5 moths after the infection [6]. These *M. tuberculosis*-exposed monkeys with transient low levels of bacillus but no evidence of active tuberculosis were treated daily for 3 months with anti-TB drugs; Isoniazid (5mg/kg) and Pyrazinamide (15mg/kg) mixing with yoga as previously described [49], and then rested subsequently for 2 months. *M. tuberculosis* infection appeared to be “cured” by the antibiotics, since repeated lung washings showed no evidence of detectable bacillus organisms; clinical follow-up demonstrated no evidence of clinical tuberculosis. Two of these monkeys were randomly assigned for anti-CD8 Ab treatment, and the other two assigned for treatment with isotype IgG at the time they were re-infected with 3000 CFU *M. tuberculosis* by aerosol.

### Anti-CD8 antibody (Ab) treatment for depletion of CD8 lymphocytes

For the CD8 Ab-treated group, each monkey was injected intravenously with depleting anti-CD8 Ab cM-T807 [37] at days 0, 3, 7 and 10 at doses of 10 mg/kg, 5 mg/kg, 5 mg/kg and 5 mg/kg, respectively. For the isotype control group, each monkey was similarly injected with human IgG at same doses. For the naïve control group, each monkey was injected similarly with saline.

### *M. tuberculosis* infection

At day 0, each monkey in BCG groups and the naïve group was infected with 3000 CFU of *M. tuberculosis* Erdman (the standard challenge stock from FDA) by the bronchoscope-guided injection of the inoculum into the right caudal lobe as previously described [29],[33]. For *M. tuberculosis*-immunized monkeys treated with anti-tuberculosis drugs, *M. tuberculosis* re-infection was introduced by aerosol challenge with about 3000 CFU *M. tuberculosis* organisms as previously described [6]. The Inhalation Exposure System used to conduct the aerosol exposure tests was enclosed within a Class III biological safety cabinet. A modified Microbiological Research Establishment type three-jet Collison nebulizer (BGI, Waltham, MA) with a precious fluid jar was used to generate a controlled delivery of *M. tuberculosis* aerosol (1–1.5 um diameter of droplets) from a PBS suspension. 10^6^ *M. tuberculosis* CFU/ml were placed in the nebulizer and monkeys were exposed for 10 min. Samples of the aerosol were collected using all-glass impingers for analyzing *M. tuberculosis* concentration (CFU/ml). The inhaled doses were determined based on the AGI concentration, sampler volume, sampling rate and respiratory minute volume of individual macaques. The inhaled doses for the individual monkeys ranged from 2800 to 3100 CFU of *M. tuberculosis*.

### Bronchoalveolar lavage (BAL)

Prior to BAL, animals were subjected to overnight or 24 h fasting, and were tranquilized i.m. with 1–2 mg/kg xylazine (Ben Venue Laboratories, Bedford, OH) and 10 mg/kg ketamine HCl. For BAL, animals also received 0.05 mg/kg atropine (Phoenix Scientific, Inc., St. Joseph, MO) i.m. as an anticholinergic and were restrained in an upright position. A pediatric feeding tube was inserted down the larynx, into the trachea through direct visualization with a laryngoscope to the level of the carina. 10 ml of saline were instilled into the trachea and immediately withdrawn and repeated a maximum of 3 times until a total of 12–15 ml BAL fluid was retrieved.

### Isolation of single cell suspensions and lymphocytes from blood and tissues

PBL were isolated from EDTA blood of the monkeys using Ficoll/diatrizoate gradient centrifugation. Lymph nodes and spleen were carefully teased to generate single-cell suspensions. Tissue pieces from lungs, livers, and kidneys were minced in RPMI medium, as previously described [29], to collect single cell suspensions (mainly lymphocytes and tissue macrophages). The single cells suspensions from these non-lymphoid organs were divided into three parts: one directly used for mycobacterial CFU counts; one directly saved as pellets for real time quantitation of *M. tuberculosis* Ag85B RNA; one subjected to isolation of lymphocytes by Ficoll/diatrizoate gradient centrifugation for flow cytometry-based analyses of T cells.

### Lymphocyte phenotying and flow cytometry analyses

Cell surface phenotyping, antibodies used for staining and flow cytometry analyses were described as we previously published [6],[33],[45]. PBMC, BAL, and tissue cells were stained with up to 5 Abs (conjugated to FITC, PE, allophycocyanin, pacific blue, and PE-Cy5 or allophycocyanin-Cy7) for at least 15 min. After staining, cells were fixed with 2% formaldehyde-PBS (Protocol Formalin, Kalamazoo, MI) prior to analysis on a CyAn ADP flow cytometer (DakoCytomation, Carpinteria, CA). Lymphocytes were gated based on forward- and side-scatters, and pulse-width and at least 40,000 gated events were analyzed using Summit Data Acquisition and Analysis Software (DakoCytomation). Absolute cell numbers were calculated based on flow cytometry data and complete blood counts performed on a hematology system (Advia 120, Siemens, Tarrytown, NY).

### Intracellular cytokine staining (ICS) measuring of mycobacterium-specific T cells

This was done as previously described [33],[36],[50]. 10^5^–10^6^ BAL cells or 10^6^ PBL plus costimulatory mAbs CD28 (1 µg/ml) and CD49d (1 µg/ml) were incubated with PPD (25ug/ml) or media alone in 200 µl final volume for 1 h at 37°C, 5% CO_2_ followed by an additional 5 h incubation in the presence of brefeldin A (GolgiPlug, BD). After staining cell-surface CD3, CD4, and CD8 for 30 min, cells were permeabilized for 45 min (Cytofix/cytoperm, BD) and stained another 45 min for IFNγ and perforin before resuspending in 2% formaldehyde-PBS.

### Bacterial colony forming units (CFU) counts

Quantitation of *M. tuberculosis* infection was done by measuring bacterial colony counts, as previously described [6],[29],[50]. To objectively measure CFU in lungs, a half of cut-sections of the right caudal lobe (the infection site), the right middle lobe or the left caudal lobe from each animal were taken for CFU determination after the extensive gross pathologic evaluation was accomplished. If there were tuberculosis lesions in the respective lobe, a half of the lung tissue containing approximately 50% lesions was taken. If no visible lesions were seen in the respective lobe, a random half of tissue was taken for evaluation. Tissue homogenates were made using a homogenizer (PRO 200, PRO Scientific INC, CT) and diluting the homogenate in sterile PBS + 0.05% Tween-80. 5-fold serial dilutions of samples were plated on Middlebrook 7H11 supplemented with OADC (10%), glycerol (0.5%), Casamino acids (0.2%; Becton Dickinson 223120), Lysine (80 mg/L; Sigma L8662), Pantothenate (24 mg/L; Sigma P5710), Polymyxin B (50,000 units/L; Sigma P1004), Amphotericin B (5 mg/L; Sigma A2942), Nalidixic acid (20 mg/L; Sigma N4382), Trimethoprim (5 mg/L; Sigma T7883) and Azlocillin (5 mg/L; Sigma A7926). The plates were then incubated in a 37°C incubator for 3 weeks, and CFU were counted. The minimal detection level of vial mycobacteria is about 2 CFU on a plate from 100 ul of 10 ml lung homogenate. The bronchoaleveolar lavage fluid was decontaminated with the MycoPrep solutions (Becton Dickinson cat 240862) and then plated on the supplemented Middlebrook 7H11.

### The real time quantitative PCR for quantitation of *M. tuberculosis* Ag85B RNA

The method and validation including detection limit, CV and relationship to bacterial colony counts were previously described [expression levels of β-actin in cells were verified and expressed as copies of *M. tb* RNA in 10 mg-equivalent tissue cells [6],[29],[36]].

### Gross pathologic analyses of TB lesions and scoring systems

Complete necropsy was done by three pathologists and 2 veterinary doctors for *M. tuberculosis*-infected monkeys as previously described [6],[29],[33]. Animals were sacrificed by intravenous barbiturate overdose, and immediately necropsied in a biological safety cabinet within a BSL-3 facility. Standard gross pathologic evaluation procedures were followed, with each step recorded and photographed. Multiple specimens from all tissues with gross lesions and remaining major organs were harvested. Specifically, each lobe of lung, bronchial, mesenteric, axillary and inguinal lymph nodes, tonsils, and other major organs were collected and labeled. Gross observations including but not limited to the presence, location, size, number and distribution of lesions were recorded. Two scoring systems were used; one system [28],[29] was used for scoring TB lesions in lungs infected by bronchoscope-guided inoculation. For each lobe of lung, granuloma prevalence was scored 0 -4 for (i) no visible granulomas, (ii) 1–3 visible granulomas, (iii) 4–10 visible granulomas, (iv) >10 visible granulomas, and (v) miliary pattern of granulomas, respectively. Granuloma size was scored 0 -3 for (i) none present, (ii) <1 – 2 mm, (iii) 3–4 mm, and (iv) >4 mm, respectively. Pulmonary consolidation or atelectasis as viewed from organ exterior and cut surfaces were scored 0 -2 for (i) absent, (ii) present focally in one lobe, and (iii) focally extensive within a lobe or involving multiple lobes. One score was also given for the presence of tuberculosis-related focal parietal pleural adhesions, pleural thickening and opacification, and pulmonary parenchymal cavitation. For hilar lymph nodes, enlargements were scored 0 -3 for (i) visible but not enlarged, (ii) visibly enlarged unilaterally (≤2cm), (iii) visibly enlarged bilaterally (≤2 cm), (iv) visibly enlarged unilaterally or bilaterally >2 cm, respectively. Tuberculosis lesions in hilar lymph nodes were scored 0 -4 for (i) no granulomas visible on capsular or cut surface, (ii) focal or multifocal, circumscribed, non-coalescing granulomas, <2mm (≥nodes), (iii) coalescing solid or caseating granulomas occupying<50% of nodal architecture (≥nodes), (iv) coalescing solid or caseating granulomas occupying >50% of nodal architecture, with residual nodal components still recognizable, and (v) complete granulomatous nodal effacement and caseation, respectively. One score was also given for tuberculosis-associated changes in other thoracic nodes. The tuberculosis lesions in each extrathoracic organ were scored similarly as each lung lobe. The pathology scoring of infected tissues was conducted in a blinded fashion by the senior pathologist D.H. The other scoring system, as described [41], was adopted for scoring >0.5 cm tubercles' involvement in each lobe of lungs infected by aerosol. Score 0 if no lung lobe involved; score 1 if <¼ of lobe involved; score 2 if ¼–½ of lobe involved; score 9 if >½ but < entire lobe involved; score 16 if entire lobe involved.

### Microscopic analyses of TB lesions and acid-fast staining

Adjacent blocks of tissues were collected and fixed in buffered 10% formalin with ionized zinc (Z-Fix™; Anatech, LTD, Battle Creek, MI), frozen with and without Optimal Cutting Temperature (OCT) compound (Sakura Finetek USA, Inc, Torrance, CA). Histologic specimens were embedded in paraffin and sectioned at 5um for routine staining with hematoxylin and eosin (H&E) and selected staining with Ziehl-Neelsen acid fast stain. The extent of lung involvement for each lung lobe was determined using digital scans of each lobe of lung to record total pixel counts on H&E stained material and specimen area measured in square cm using Image-Pro Plus software (MediaCybernetics, Silver Spring, MD). Granulomas were objectively compared for the size, type of granuloma (caseous, solid, suppurative, or mixed), distribution pattern (focal, multifocal, coalescing, and invasive), and cellular composition (absence or presence with degree of lymphocytic cuff, mineralization, fibrosis, multinucleated giant cells, and epithelioid macrophages) between and within monkey groups.

### Statistical analysis

The multivariate analysis of variance (ANOVA) and nonparametric student *t* test were used, as previously described [29],[33],[36], to statistically analyze the data for differences in T cell numbers, *M. tuberculosis* burdens, gross pathology scores or specific lesion sizes between CD8 Ab-treated and isotype IgG-treated or naïve control groups.
